# The prevalence and natural history of hepatic cysts examined by ultrasound: a health checkup population retrospective cohort study

**DOI:** 10.1038/s41598-022-16875-z

**Published:** 2022-07-27

**Authors:** Kota Tsuruya, Yasuhiro Nishizaki, Masayuki Tatemichi, Yusuke Mishima, Yoshimasa Shimma, Yoshitaka Arase, Shunji Hirose, Koichi Shiraishi, Tatehiro Kagawa

**Affiliations:** 1 Division of Gastroenterology and Hepatology, Department of Internal Medicine, Tokai University School of Medicine, 143 Shimokasuya, Isehara, 259-1193 Japan; 2 Department of Clinical Health Science, Tokai University School of Medicine, Isehara, Japan; 3 Department of Preventive Medicine, Tokai University School of Medicine, Isehara, Japan

**Keywords:** Hepatology, Risk factors

## Abstract

The prevalence of hepatic cysts in the general population and their natural history are largely unknown. This study aimed to assess the prevalence and natural history of hepatic cysts by investigating health checkup participants. Ultrasonographic data of health checkup participants (n = 38,842) were retrospectively evaluated to calculate its prevalence. In addition, we assessed the changes in the size and characteristics of hepatic cysts over 10 years (n = 7709). We found the prevalence of hepatic cysts was 21.9%. Older age, female sex, and presence of kidney cysts or pancreatic cysts were associated with the occurrence of hepatic cysts. Younger age, female sex, and the existence of multiple hepatic cysts were associated with cyst enlargement. Among 126 individuals who had hepatic cysts with a diameter of 30 mm or larger at the first visit, two (1.6%) required treatment. Remain 124 cases showed four patterns: 44 cases with enlargement, 47 stable, 11 regression after enlargement, and 22 regression. Hyperechoic fluid inside the cysts was observed in 54.5% (18 of 33), which was significantly higher than 6.6% (6 of 91) of the non-regression (OR = 17.0). The appearance of intracystic hyperechoic fluid by ultrasound may predict subsequent regression of the hepatic cyst.

## Introduction

Hepatic cysts are fluid-filled cavities lined by a single-layered cuboidal or columnar biliary epithelium in the liver^1–3^ and are a relatively common condition encountered in daily practice. A majority of hepatic cysts are found incidentally on liver imaging, such as abdominal ultrasonography (US), computed tomography (CT), or magnetic resonance imaging (MRI). Hepatic cystic lesions are heterogeneous clusters; most are simple hepatic cysts, but some could be malignant tumors, such as cystadenocarcinoma^4–7^. Simple hepatic cysts are believed to be congenital biliary developmental aberrations. During embryogenesis, aberrant intrahepatic bile ducts develop and dilate to form hepatic cysts^4^.

Hepatic cysts are generally asymptomatic and do not require special treatment^8,9^. However, complications such as infection or rupture may cause fever or abdominal pain. In addition, large hepatic cysts occasionally cause unpleasant symptoms, such as abdominal distention, jaundice, portal hypertension, and leg edema by compressing neighborhood organs or hepatic vasculature^10–12^. Polycystic liver disease (PLD), where most of the liver is replaced by cysts, can progress to liver failure and requires the patient to undergo liver transplantation^13^.

The prevalence of simple hepatic cysts has been shown to be increasingly common with age^14–19^. In addition, according to the study of autosomal dominant polycystic kidney disease (ADPKD), the total hepatic cyst volume increases with age^18^. Since simple hepatic cysts are usually asymptomatic, there are few reports on their natural history^19,20^. A study of 607 adult volunteers with a median follow-up of 4.8 years showed that both the mean hepatic cyst size and the number of cysts increased with time on MRI^19^. However, some cysts revealed a regression trend^20^. The mechanisms of cyst enlargement or regression are still unclear. In this study, we aimed to elucidate the prevalence and assess the change in size of hepatic cysts in a large population cohort using health checkup participants over a span of 10 years.

## Results

### Participant characteristics

A total of 38,842 individuals underwent 189,602 health checkups, including abdominal US, between July 2005 and February 2018 at the Health Check-up Center of Tokai University. The number of health checkups per person was 4.68 ± 3.29 (mean ± SD, range 1–25) times. Of these, 7709 individuals received health checkups for over 10 years. There were a greater number of males (56.7%) in this cohort (n = 38,842) and the mean ± SD age was 53.3 ± 11.6 years. Of these, 8487 (21.9%) were diagnosed with hepatic cysts at their first health checkup, with 11.9% having solitary cysts and 10.0% having multiple cysts. The size of the largest cyst was ≤ 10 mm, 11–30 mm, 31–50 mm, and ≥ 51 mm in 4433 (11.4%), 3379 (8.7%), 467 (1.2%), and 208 (0.5%) individuals, respectively. Kidney cysts and pancreatic cysts were found in 7062 (18.2%) and in 356 (0.9%) individuals, respectively.

### Hepatic cyst features

**Figure 1:**
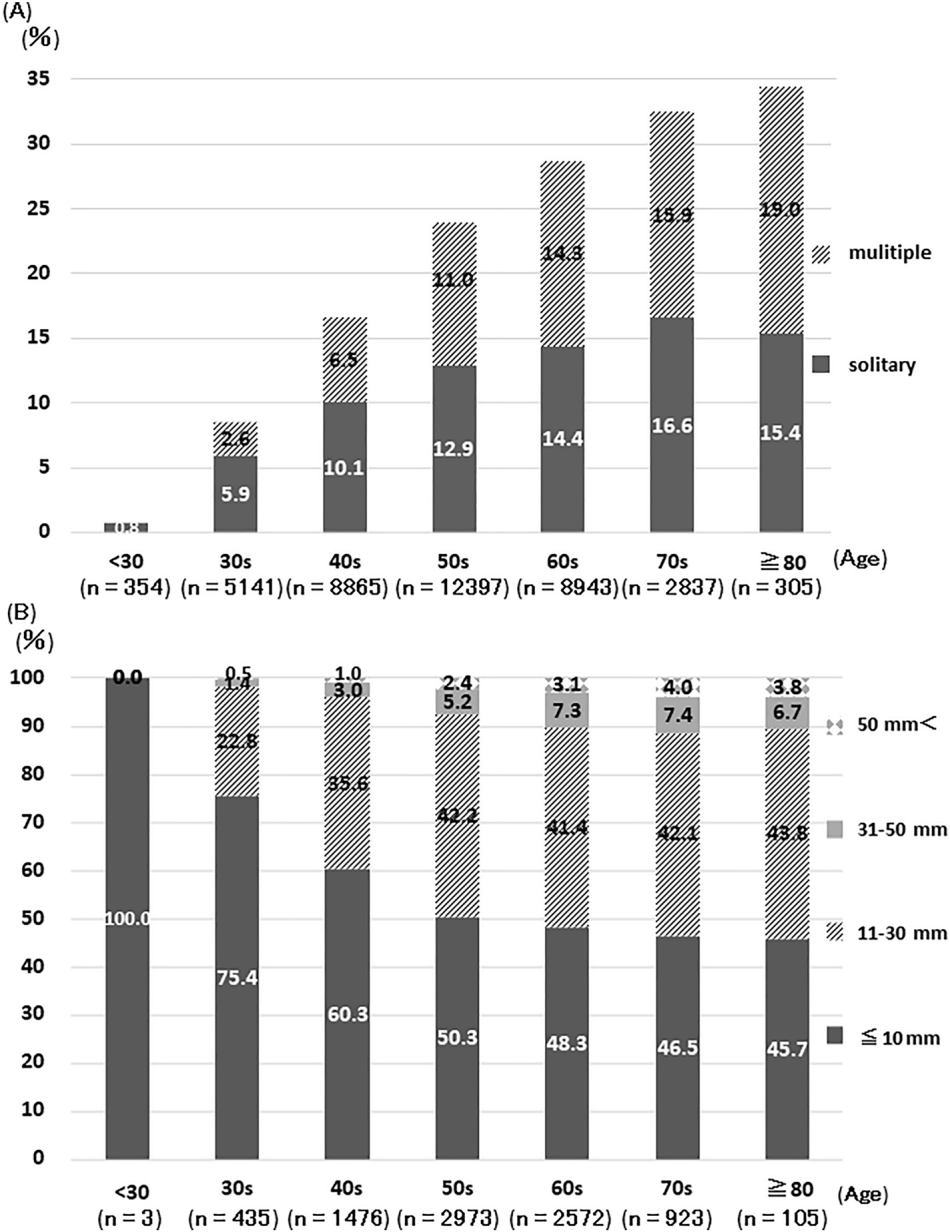
(**A**) Prevalence of hepatic cysts at first health checkup by age group. Multiple cysts was defined as two or more hepatic cysts. (**B**) The maximum hepatic cyst size and age group.

Of 38,842 individuals, the prevalence of hepatic cysts increased with age. The prevalence was 0.8%, 8.5%, 16.6%, 23.9%, 28.7%, 32.5%, and 34.4% for those less than 30 years, between 30–39 years, 40–49 years, 50–59 years, 60–69 years, 70–79 years, and 80 years or older, respectively (Fig. 1A). Surprisingly, the chance of detecting hepatic cysts increased by more than 30-fold when comparing individuals under 30 years to those over 80 years. The percentage of individuals with multiple (two or more) cysts also increased with age. Hepatic cysts exhibited a trend of growing in size with increased age (Fig. 1B). Cysts larger than 10 mm in diameter were not found in individuals under 30 years. On the other hand, 10.6% of individuals over 60 years of age had a least one cyst larger than 30 mm. Except for the caudate lobe, there were no significant differences in the location of the maximum sized cyst among liver segments (Supplementary Table 1). There was an increased chance of additional hepatic or kidney cysts with an increase in size of the hepatic cyst (Table 1).

**Table 1:** Demographics and ultrasonographic findings according to hepatic cyst size.

Variables	None	≤ 10 mm	11–30 mm	31–50 mm	50 mm <
	n = 30,355	n = 4433	n = 3379	n = 467	n = 208
Age (years), mean ± SD	52.22 ± 11.66	56.09 ± 10.72	58.25 ± 9.79	60.73 ± 8.96	61.53 ± 9.08
Female, n (%)	13,070 (43.1)	1786 (40.3)	1594 (47.2)	262 (56.1)	119 (57.2)
Multiple hepatic cysts, n (%)*	–	1273 (28.8)	2046 (60.6)	367 (78.8)	183 (88.0)
Kidney cyst, n (%)	4944 (16.3)	1060 (23.9)	850 (25.2)	137 (29.3)	71 (34.1)
Pancreatic cyst, n (%)	231 (0.8)	60 (1.4)	53 (1.6)	11 (2.4)	1 (0.5)
Splenic cyst, n (%)	42 (0.1)	6 (0.1)	5 (0.1)	1 (0.2)	0

*SD* standard deviation.

*Number information was missing in 12 cases (8: ≤ 10 mm, 3: 11–30 mm, and 1: 31–50 mm).

We analyzed the variables associated with the occurrence of hepatic cysts. Age, sex, kidney cyst, and pancreatic cyst was significantly different between groups with and without hepatic cyst, but not splenic cyst. Multivariate analysis revealed that increasing age (odds ratio [OR] 1.04, 95% confidence interval [CI] 1.04–1.04, p < 0.001), female sex (OR = 1.13, 95% CI 1.07–1.18, p < 0.001), and presence of kidney cysts (OR = 1.36, 95% CI 1.28–1.45, p < 0.001) and pancreatic cysts (OR = 1.33, 95% CI 1.06–1.66, p = 0.013) were significant variables associated with the occurrence of hepatic cysts (Table 2). Overall, the prevalence of hepatic cysts was significantly higher in females, especially those in the 50–59 years range. Although not significant, the prevalence was higher in males aged 70 years or older (Supplementary Fig. 1).

**Table 2:** Clinical characteristics in individuals with and without hepatic cysts.

Variables	Hepatic cyst (−)	Hepatic cyst (+)	p-value	Multivariate analysis by logistic regression method		
	n = 30,355	n = 8487		OR	95% CI	p-value
Age (years), mean ± SD	52.2 ± 11.7	57.3 ± 10.3	< 0.001^a^	1.04	1.04–1.04	< 0.001
Female, n (%)	13,070 (43.1)	3761 (44.3)	0.039^b^	1.13	1.07–1.18	< 0.001
Kidney cyst (+), n (%)	4944 (16.3)	2118 (25.0)	< 0.001^b^	1.36	1.28–1.45	< 0.001
Pancreatic cyst (+), n (%)	231 (0.8)	125 (1.5)	< 0.001^b^	1.33	1.06–1.66	0.013
Splenic cyst (+), n (%)	42 (0.001)	12 (0.001)	0.947^b^	–	–	–

^a^Student *t* test, ^b^Chi-squared test.

*SD* standard deviation, *OR* Odds ratio, *CI* confidence interval.

In addition, to identify the associated factors in blood chemistry logistic analysis was done. And results show that significant factors were lower serum albumin levels (OR = 0.78, 95% CI 0.71–0.86, p < 0.001), lower γ-glutamyltranspeptidase (GGT) levels, lower uric acid levels, higher low density lipoprotein (LDL)-cholesterol levels, lower glucose levels, and higher systolic blood pressure (Supplementary Table 2). When the participants were divided into three groups according to serum albumin levels, the prevalence of hepatic cysts was significantly higher in the group with low albumin levels: 17.5% for albumin ≥ 4.6 g/dl, 22.0% for albumin 4.3–4.5 g/dl, and 25.1% for albumin ≤ 4.2 g/dl, respectively (Supplementary Fig. 2).

### Hepatic cysts size change over 10 years

We analyzed changes in the size of hepatic cysts in 7709 individuals who received a health checkup 10 years after their initial checkup. Of these, 1589 (20.6%) were diagnosed with hepatic cysts at their first health checkup. The mean ± SD period between the first and last checkup was 11.3 ± 0.6 (range 10.0–12.6) years. The changes in diameter of the hepatic cysts, demographics, and US findings of the participants at their first and last visits were analyzed (Supplementary Table 3). Cyst enlargement was observed in 3.3% (53 of 1589) of individuals. Table 3 shows the factors associated with the enlargement of cyst. Age, sex, and multiple hepatic cysts were significantly different between two groups. Age in enlargement group was younger than that in non-enlargement group. Multivariate analysis showed that age (OR = 0.95, 95% CI 0.92–0.98, p = 0.004), female sex (OR = 2.25, 95% CI 1.27–4.01, p = 0.006), and existence of multiple hepatic cysts (OR = 3.00, 95% CI 1.68–5.38, p < 0.001) were independent risk factors for cyst enlargement (Table 3). We found no association between laboratory variables and cyst enlargement (data not shown).

**Table 3:** Demographic and clinical characteristics in individuals with enlarged hepatic cysts.

Variables	Non-enlargement group	Enlargement group	p-value	Multivariate analysis by logistic regression method		
	n = 966	n = 53		OR	95% CI	p-value
Age (years), mean ± SD	55.9 ± 8.9	52.5 ± 7.4	0.008^a^	0.95	0.92–0.98	0.004
Female, n (%)	394 (40.8)	33 (62.3)	0.002^b^	2.25	1.27–4.01	0.006
Multiple hepatic cysts, n (%)*	360 (37.4)	33 (62.3)	< 0.001^b^	3.00	1.68–5.38	< 0.001
Kidney cyst, n (%)	188 (19.5)	8 (15.1)	0.432^b^	–	–	–
Pancreatic cyst, n (%)	53 (5.5)	0		–	–	–
Splenic cyst, n (%)	1 (0.01)	0		–	–	–

^a^Student *t* test, ^b^Chi-squared test.

*OR* odds ratio, *CI* confidence interval, *SD* standard deviation.

Cyst enlargement was defined as a transition from ≤ 10 to ≥ 31 mm, or from 11–30 mm to ≥ 51 mm.

The non-enlargement group was defined as stable in ≤ 10 mm, a transition from ≤ 10 mm to no cyst, stable in 11–30 mm, and a transition from 11–30 mm to ≤ 10 mm or no cyst.

*Number information was missing in 3 cases.

### Detailed analysis of hepatic cysts larger than 31 mm between the first health checkup and 10 years later

We evaluated the change in hepatic cyst size in 126 individuals who had cysts larger than 31 mm at their first health checkup and received checkups for over 10 years. During the observation period, two individuals underwent therapeutic intervention for hepatic cysts. A 64-year-old man complained of abdominal distension due to enlargement of his hepatic cyst from 125 to 140 mm in 9 years. The other patient, a 57-year-old female, suffered from infection of her 58 mm hepatic cyst. Both recovered by cyst aspiration followed by sclerotherapy.

**Figure 2:**
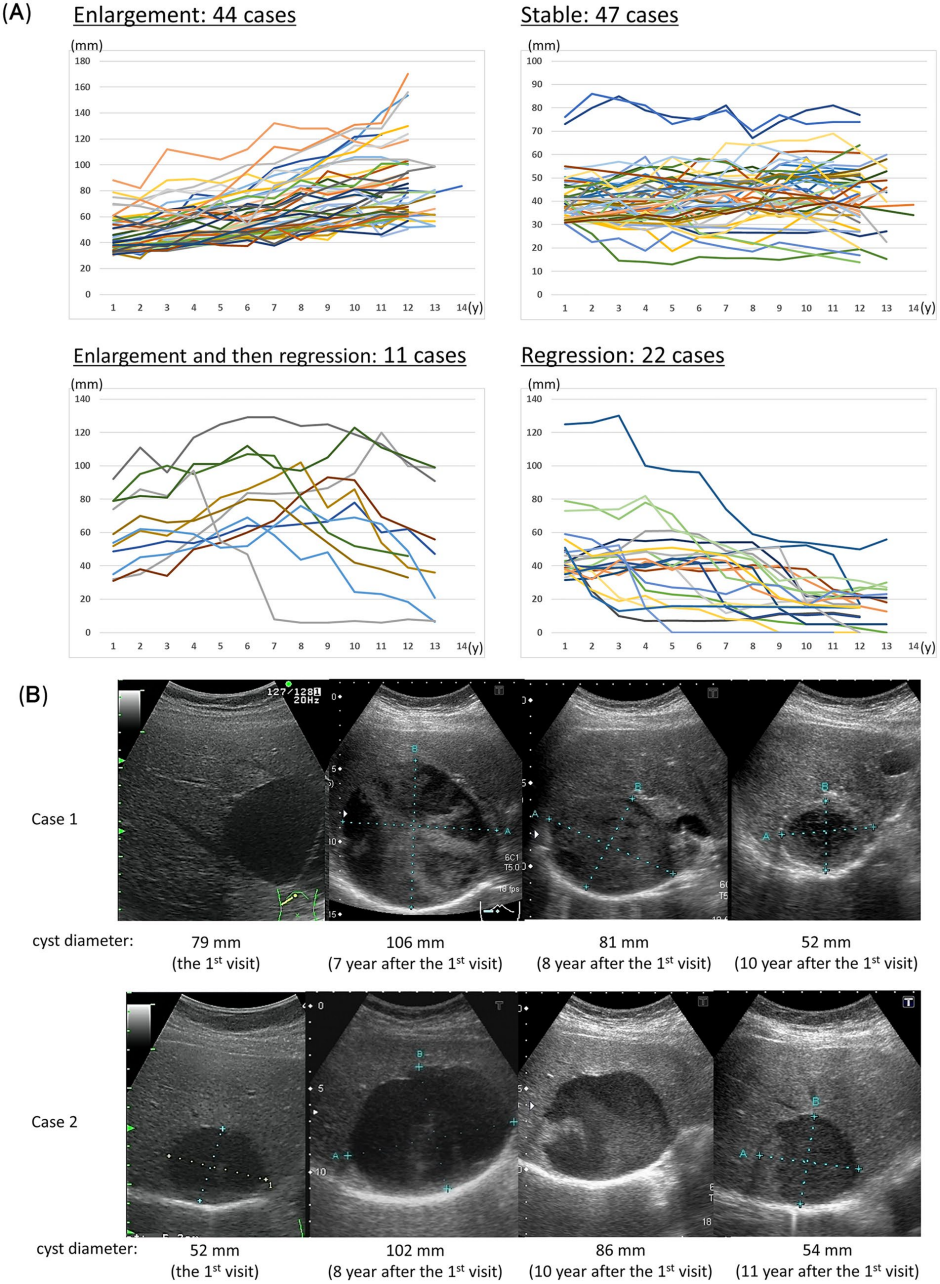
(**A**) Changes 10 years later in the diameter of hepatic cysts that were ≥ 31 mm at the first health checkup. (**B**) Ultrasonographic Imaging. Case 1 represents a regression case where intracystic hyperechoic fluid was observed in the seventh year followed by regression in cyst size. Case 2 represents a regression case where intracystic hyperechoic fluid was observed in the tenth year followed by regression in cyst size.

Finally, we analyzed the remaining 124 cases. The average cyst size was 45.4 ± 15.6 and 54.0 ± 33.9 (mean ± SD) mm at the first and last checkup, respectively. Four patterns such as enlargement, stable, enlargement and then regression, and regression were observed in 44, 47, 11, and 22 cases, respectively (Fig. 2A). Cases with cyst regression was observed in 26.6% (33 of 124) individuals. Interestingly, hyperechoic fluid inside the cysts emerged (Fig. 2B) in 54.5% (18 of 33) of the regression group, which was significantly higher than 6.6% (6 of 91) of the non-regression group (OR = 17.0, 95% CI = 5.8–49.8, p < 0.001). There were no significant differences in age, sex, maximum cyst diameter, or the presence of kidney cysts between the regression and non-regression groups (Supplementary Table 4).

## Discussion

In the present study, we analyzed the prevalence, clinical findings, and long-term course of hepatic cysts in health checkup participants. The prevalence of hepatic cysts has been reported in various studies. However, this prevalence highly differs depending on study size, patient population, and diagnostic modalities or techniques. In our cohort, the prevalence of hepatic cysts diagnosed by US was 21.9%. This rate was higher than the previously reported rates ranging from 2.5 to 5.8% in studies that also utilized US^14,15,21,22^. This difference may be attributable to the difference in performance of the US devices or techniques. On the other hand, when CT or MRI were used, the prevalence was 17.8%^17^ or 71.0%^19^, respectively. Thus, hepatic cysts appear to be more prevalent than previously thought. The prevalence of hepatic cysts in women in our cohort was significantly higher in accordance with previous studies^14,22^. However, when we analyzed the prevalence by age group, a significant sex difference was only observed in individuals in their 50s. However, the 50s group was the largest population in our cohort and this may have affected the overall sex difference. Although the age distribution of participants was relatively similar to our cohort, Blum et al.^19^ reported that hepatic cysts were more common in men. Hence, further elucidation of the mechanisms of hepatic cyst formation, such as differences in genetic background, is needed.

We found that individuals with hepatic cysts had a higher possibility of accompanying kidney or pancreatic cysts. So far, no studies have demonstrated the interrelationship among these different cysts. Hepatic cysts are often associated with polycystic kidney disease^18^, which is due to genetic abnormalities of the renal tubular and biliary epithelial cell membrane proteins, such as PKD1, PKD2, or PKHD1. As these proteins are involved in cilia formation, the diseases caused by the lack of their function are called ciliopathy^23,24^. In addition, ADPKD is associated with a high incidence of pancreatic cysts and hepatic cysts^25^. Although the mechanism of how simple cysts emerge is unknown, concomitant occurrence of cysts in multiple organs suggests the involvement of a genetic background that leads to cilia malfunction.

Our study also revealed that the prevalence of hepatic cysts increased with age as previously reported^14–19^. This observation was supported by our results that the percentage of individuals with hepatic cysts doubled (from 20.6 to 40.7%) in those individuals who received repeated health checkups for more than 10 years after their first checkup. Interestingly, older age was also associated with kidney and pancreatic cysts^17,26,27^. Therefore, it might be generalized that risk of cyst occurrence increases with aging. We also found that the presence of hepatic cysts was associated with the following laboratory variables; lower serum albumin, lower serum GGT, lower serum uric acid, higher LDL-cholesterol, lower glucose, and lower systolic blood pressure. Among these, the albumin level was a strong predictor for hepatic cysts (Supplementary Fig. 2). A recent study identified lower glucose levels, lower uric acid levels, lower triglyceride levels, a lower waist-to-hip ratio, and lower systolic blood pressure as variables associated with the occurrence of hepatic cysts^19^. The implication of these factors in hepatic cyst development is unclear and requires further study.

Only a few studies have examined the natural course of hepatic cysts. We analyzed the change of cyst size in individuals who had a cyst with a diameter of 31 mm or larger at their first checkup. They grew from 45.4 to 54.0 mm on average in 11.3 years. In agreement with our study, a study utilizing MRI revealed that the maximum cyst size increased on average from 13.7 to 15.3 mm in 4.8 years^19^. A study of PLD showed that being a young woman was associated with hepatic cyst enlargement, suggesting a hormonal influence^28^. We also confirmed that diagnosis of hepatic cysts at a younger age, female sex, and existence of multiple hepatic cysts were independent risk factors for cyst enlargement.

Notably, on the contrary, cysts occasionally regress. For the first time, we demonstrated that the appearance of intracystic hyperechoic fluid anticipated cyst regression in 18 of 24 (75%) cysts. Echogenic materials inside the cyst suggest they are complicated cysts, such as those with hemorrhage or infection. Khono and his colleagues analyzed surgically resected hemorrhagic hepatic cysts and concluded that the decrease in cyst size after hemorrhage resulted from the absorption and organization process of hematoma^29^. Therefore, when we find hyperechoic fluid inside cysts, they may regress in the future. However, there exist cases that require special attention. Arterial hemorrhage into the cyst can cause a shock^30^ and cystadenocarcinoma may cause hemorrhage into the cyst^31,32^. When these situations are suspected, other modalities, such as contrast-enhanced CT or MRI, are recommended^33^. We did not find any patients with cystadenocarcinoma in our cohort. Only 2 out of 35 (5.7%) individuals with hepatic cysts larger than 51 mm at their first visit and received repeated checkups over 10 years required treatment due to abdominal distention and infection. Both patients successfully underwent cyst aspiration followed by sclerotherapy^34^. Size, as well as cyst location, is associated with causing symptoms.

Our study had several limitations. First, this is a retrospective, single-center study. Second, the cyst diameter was measured by US, which is prone to greater measurement error and technician variability than MRI or CT. Third, our cohort might include patients with genetic diseases such as PCLD or ADPKD, although subjects were health checkup participants. The natural history of hepatic cysts in these patients may differ from that of simple cysts. Finally, there is a possibility of selection bias because individuals who became symptomatic were likely to cease receiving health checkups. As our study was based upon health checkups, these patients would be missed from the analysis.

In conclusion, the occurrence of hepatic cysts was associated with older age, female sex, and the presence of kidney or pancreatic cysts. Hepatic cysts were more likely enlarged in younger individuals at diagnosis, the female sex, and those with multiple hepatic cysts. In addition, appearance of intracystic hyperechoic fluid might predict subsequent regression of the cysts.

## Methods

### Study design

This is a retrospective single-center study to investigate the prevalence and natural history of hepatic cysts by analyzing consecutive medical records performed as a health checkup at the Health Check-up Center of Tokai University between July 2005 and February 2018. We obtained participant information, such as age, sex, body mass index, laboratory findings, and blood pressure.

### Ultrasound examination

US was performed by clinical laboratory technicians under supervision of physicians belonging to the Clinical Health Science Department using the Nemio 20 (Toshiba Medical Systems Corporation, Otawara, Japan), Nemio XG (Toshiba), Xario (Toshiba), or ProSound SSD-3500 (Hitachi Aloka Medical, Tokyo, Japan) equipped with convex probes. Our US facility, certified by ISO 15189, maintains US performance skills by providing regular training to technicians and physicians. Most of them are certified as registered medical sonographers of the Japan Society of Ultrasonics in Medicine. Abdominal US screenings were performed using the intercostal, subcostal, longitudinal, and transverse scanning in a fan and slide motion that allowed evaluation of the entire liver parenchyma, intrahepatic bile ducts, gallbladder, pancreas, kidneys, and spleen. The diagnosis of hepatic cysts was made based upon the presence of anechoic circular or oval lesions with smooth borders and posterior acoustic enhancement^35^. The size of the hepatic cyst was determined according to the largest diameter of each cyst and classified into four categories; ≤ 10 mm, 11–30 mm, 31–50 mm, and ≥ 51 mm. When the examiners found a cyst, they described the number of cysts (single or multiple), cyst size category, and location (liver segment) in the final reports which were reviewed by another technician and physician. Information on echoic or mobile materials was also documented if they were present inside the cyst.

### Definition of cyst size change

We also analyzed the change in the cyst size category between the first and last health checkup in subjects who received checkups for over 10 years. In order to exclude cases with only a subtle change, cyst enlargement was defined as either a two or three-stage size up (a transition from ≤ 10 to ≥ 31 mm or ≥ 51 mm or a transition from 11–30 mm to ≥ 51 mm). The non-enlargement group was defined as stable in ≤ 10 mm, a transition from ≤ 10 mm to no cyst, stable in 11–30 mm, and a transition from 11–30 mm to ≤ 10 mm or no cyst. Individuals who had a cyst with a diameter of 30 mm or greater at the first visit and underwent a checkup over 10 years later were analyzed in more detail in terms of cyst size and US findings at each visit. In this analysis, cyst enlargement and regression was defined as a 20 mm or more growth or shrinkage in diameter, respectively.

### Statistical analysis

Demographic variables were shown as the mean ± standard deviation (SD). Numerical and categorical variables were compared between two groups using Student’s *t* test and Chi-squared test, respectively. Odds ratio was calculated in 2 × 2 tables. The independent association of presence or enlargement of hepatic cysts with age and cysts of other organs was evaluated by multiple logistic regression analysis. First variables with a p-value < 0.05 were entered in the model. Second, to identify independent factors of blood chemistry data multivariate regression analyses were performed using forward method. A p-value < 0.05 was considered statistically significant. All analyses were performed using SPSS Statistics Ver.26 (IBM Corp., Armonk, NY).

### Ethical considerations

This research was conducted with the approval of the Ethics Review Board of the Tokai University School of Medicine (18R-022) and was conducted in accordance with the Declaration of Helsinki. All participants gave written informed consent to the use of their health records for analysis.

## Supplementary Information

Supplementary Information.

## Data Availability

The datasets used and analyzed during the current study available from the corresponding author on reasonable request.
