# Supplementary material for: The prevalence and natural history of hepatic cysts examined by ultrasound: a health checkup population retrospective cohort study
Source: Sci Rep. 2022 Jul 27;12:12797. doi: 10.1038/s41598-022-16875-z (PMC9329350; doi:10.1038/s41598-022-16875-z)
Supplement: Supplementary file 1 — Supplementary Information. [file 41598_2022_16875_MOESM1_ESM.docx]

**Supplementary Materials**

The prevalence and natural history of hepatic cysts examined by ultrasound: A health checkup population retrospective cohort study

Kota Tsuruya^1^, Yasuhiro Nishizaki^2^, Masayuki Tatemichi^3^, Yusuke Mishima^1^, Yoshimasa Shimma^1^, Yoshitaka Arase^1^, Shunji Hirose^1^, Koichi Shiraishi^1^, and Tatehiro Kagawa^1^

*^1^ Division of Gastroenterology and Hepatology, Department of Internal Medicine, ^2^ Department of Clinical Health Science, ^3^ Department of Preventive Medicine, Tokai University School of Medicine*

**Supplementary Figure Legends (page 2)**

**Supplementary Figures (page 3-4)**

**Supplementary Tables (page 5-9)**

**Supplementary Figure Legends**

**Supplementary Figure 1:**

Hepatic cyst occurrence according to sex. *P <0.001.

**Supplementary Figure 2:**

Hepatic cyst occurrence according to serum albumin level. The serum albumin level was missing for 2 cases. *P <0.001.

**Supplementary Figures**

**Supplementary Figure 1**


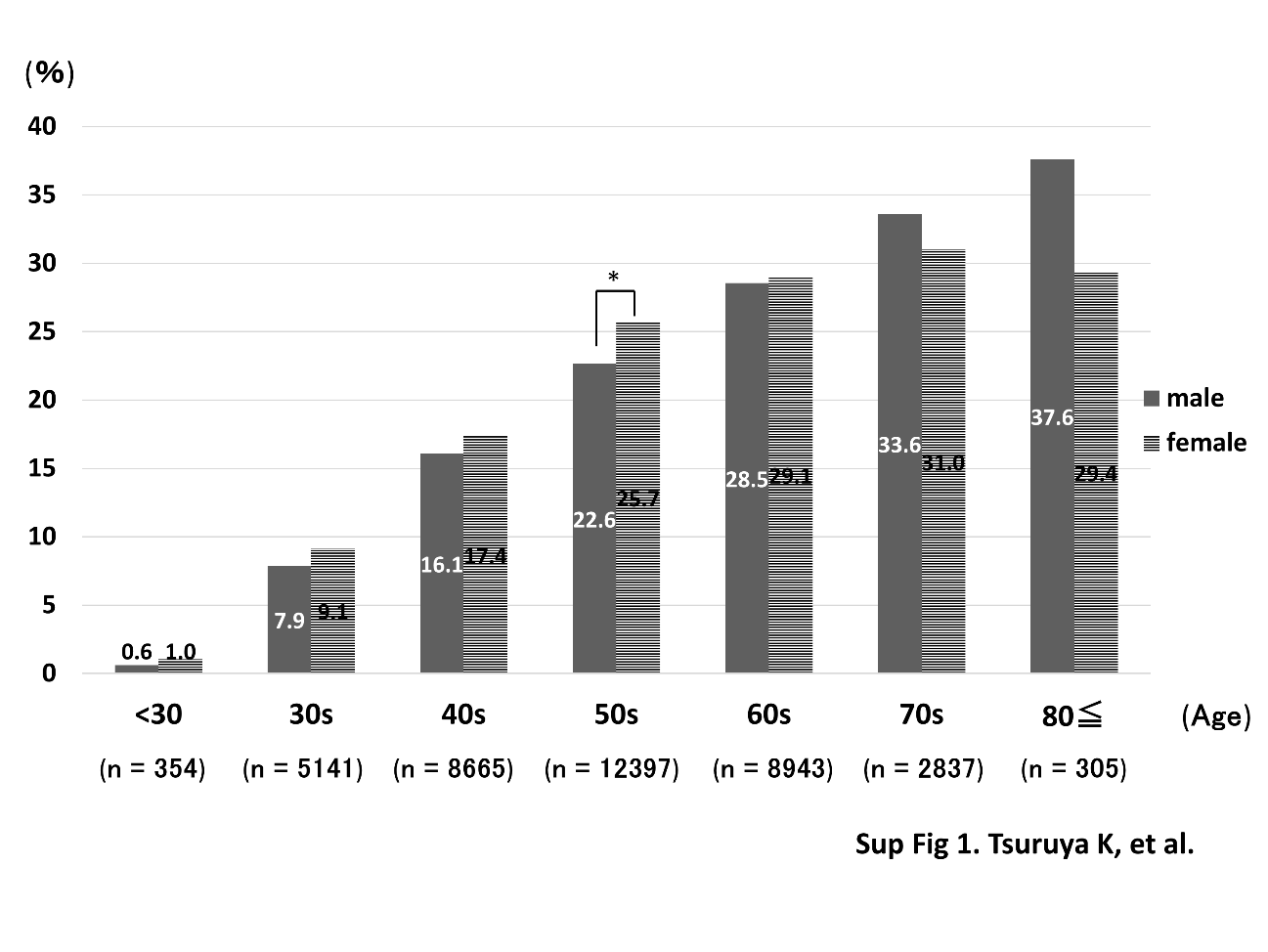


**Supplementary Figure 2**


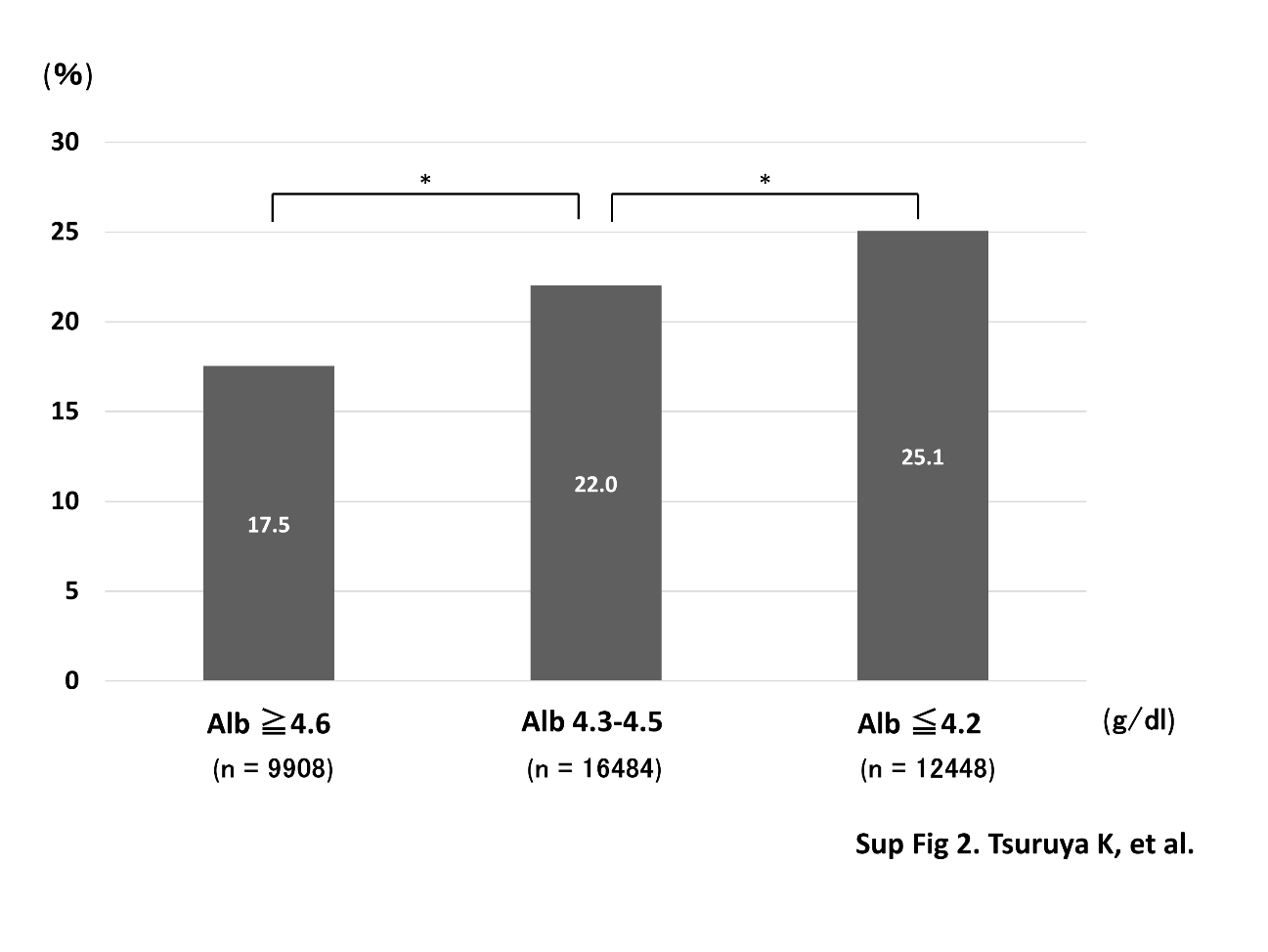


**Supplementary Tables**

**Supplementary Table 1**

Hepatic cyst location

| Location | Solitary cyst, n (%) | Largest cyst in multiple cysts, n (%) |
| --- | --- | --- |
| S1  S2  S3  S4  S5  S6  S7  S8 | 23 (0.5)  707 (15.4)  639 (13.6)  692 (15.0)  755 (16.4)  610 (13.3)  449 (9.8)  728 (15.8) | 13 (0.3)  603 (15.6)  542 (14.0)  626 (16.2)  448 (11.6)  468 (12.1)  441 (11.4)  723 (18.7) |

Location and number information were missing in 8 and 12 cases, respectively.

**Supplementary Table 2**

Demographic, clinical characteristics, and laboratory data in individuals with and without hepatic cysts

| Variables | Hepatic cyst (-)  n = 30355 | Hepatic cyst (+)  n = 8487 | P-value | Multivariate analysis by logistic　regression method | | |
| --- | --- | --- | --- | --- | --- | --- |
|  |  |  |  | OR | 95% CI | P-value |
| Age (years), mean±SD | 52.2 ± 11.7 | 57.3 ± 10.3 | <0.001 ^a^ | 1.038 | 1.036-1.041 | <0.001 |
| Female, n (%) | 13070 (43.1) | 3761 (44.3) | 0.039 ^b^ | 1.086 | 1.021-1.156 | 0.009 |
| kidney cyst (+), n (%) | 4944 (16.3) | 2118 (25.0) | <0.001 ^b^ | 1.340 | 1.260-1.425 | <0.001 |
| Pancreatic cyst (+), n (%) | 231 (0.8) | 125 (1.5) | <0.001 ^b^ | 1.323 | 1.055-1.658 | 0.015 |
| Splenic cyst (+), n (%) | 42 (0.001) | 12 (0.001) | 0.947 ^b^ | - | - | - |
| BMI (kg/m^2^), mean ± SD | 22.8 ± 3.3 | 22.7 ± 3.1 | 0.001 ^a^ | 0.999 | 0.990-1.008 | 0.835 |
| Albumin (g/dl), mean ± SD | 4.38 ± 0.28 | 4.33 ± 0.27 | <0.001 ^a^ | 0.782 | 0.709-0.863 | <0.001 |
| ALT (U/L), mean ± SD | 23.0 ± 24.4 | 21.4 ± 16.4 | <0.001 ^a^ | 1.000 | 0.999-1.002 | 0.824 |
| GGT (U/L), mean ± SD | 38.3± 46.5 | 33.4 ± 42.7 | <0.001 ^a^ | 0.998 | 0.997-0.998 | <0.001 |
| T-Bil (mg/dl), mean ± SD | 0.82 ± 0.33 | 0.80 ± 0.32 | <0.001 ^a^ | 0.947 | 0.874-1.027 | 0.188 |
| Uric acid (mg/dl), mean ± SD; | 5.42 ± 1.36 | 5.35 ± 1.34 | <0.001 ^a^ | 0.960 | 0.938-0.983 | 0.001 |
| Triglycerides (mg/dl), mean ± SD | 111.9 ± 77.3 | 108.1 ± 66.5 | <0.001 ^a^ | 1.000 | 1.000-1.000 | 0.721 |
| LDL-Chol (mg/dl), mean ± SD | 125.3 ± 30.9 | 127.3 ± 30.0 | <0.001 ^a^ | 1.001 | 1.000-1.002 | 0.005 |
| Glucose (mg/dl), mean ± SD | 101.2 ± 18.8 | 100.1 ± 15.1 | <0.001 ^a^ | 0.990 | 0.988-0.992 | <0.001 |
| HBs-Ag positive,  n (%) | 314 (1.0) | 107 (1.3) | 0.076 ^b^ | - | - | - |
| HCV-Ab positive,  n (%) | 302 (1.0) | 115 (1.5) | 0.005 ^b^ | 1.118 | 0.895-1.397 | 0.325 |
| Systolic BP (mmHg), mean ± SD | 122.4 ± 20.1 | 124.3 ± 20.2 | <0.001 ^a^ | 1.002 | 1.000-1.003 | 0.011 |

^a^ Student-t test, ^b^ chi-squared test

OR; odds ratio, CI; confidence interval, SD; standard deviation, BMI; body mass index, ALT; alanine transaminase, AST; aspartate transaminase, GGT; gamma-glutamyltransferase, T-Bil; Total bilirubin, LDL-Chol; low density lipoprotein cholesterol, HBs-Ag; hepatitis B surface antigen, HCV-Ab; hepatitis C antibody, BP; blood pressure.

**Supplementary Table 3**

Changes in hepatic cyst size between the first and final health checkups

| First visit | | Last visit | |
| --- | --- | --- | --- |
| size | n | size | n (%) |
| None | 6120 | None  ≦10 mm  11–30 mm  31–50 mm  50 mm＜ | 4438 (72.5)  1316 (21.5)  354 (5.8)  11 (0.002)  1 (0.00002) |
| ≦10 mm | 776 | None  ≦10 mm  11–30 mm  31–50 mm  50 mm＜ | 108 (13.9)  338 (43.6)  315 (40.6)  14 (1.8)  1 (0.001) |
| 11–30 mm | 687 | None  ≦10 mm  11–30 mm  31–50 mm  50 mm＜ | 23 (3.3)  94 (13.7)  403 (58.7)  129 (18.8)  38 (5.5) |
| 31–50 mm | 91 | None  ≦10 mm  11–30 mm  31–50 mm  50 mm＜ | 1 (1.1)  5 (5.5)  17 (18.7)  27 (29.7)  41 (45.1) |
| 50 mm＜ | 35 | None  ≦10 mm  11–30 mm  31–50 mm  50 mm＜ | -  -  7 (20.0)  4 (11.4)  24 (68.6) |

**Supplementary Table 4**

Demographics and ultrasonographic findings in individuals whose hepatic cysts were regressed

| Variables | Non-regression group  n = 91 | Regression group  n = 33 | Univariate analysis | | |
| --- | --- | --- | --- | --- | --- |
|  |  |  | OR | 95% CI | P-value |
| Age (years) at first visit, mean±SD | 59.0±8.1 | 56.1±8.1 |  |  | 0.089 |
| Female, n (%) | 44 (48.4) | 20 (60.6) |  |  | 0.230 |
| Multiple hepatic cysts, n (%) | 73 (80.2) | 26 (78.8) |  |  | 0.861 |
| Kidney cyst, n (%) | 25 (27.5) | 5 (15.2) |  |  | 0.471 |
| Maximum cyst diameter under observation, cm, median (IQR) | 60.6 (47.5 – 84.0) | 56.0 (45.4 – 87.5) |  |  | 0.892 |
| Appearance of intracystic hyperechoic fluid, n (%) | 6 (6.6) | 18(54.5) | 17.00 | 5.80-49.79 | <0.001 |

SD; standard deviation, IQR; interquartile range. Hepatic cyst regression was defined as 2 cm and more shrinkage in diameter over 10 years.
